# Case for diagnosis. Noninfectious suppurative panniculitis induced by mesotherapy with deoxycholate^☆^^☆☆^

**DOI:** 10.1016/j.abd.2019.02.003

**Published:** 2019-10-24

**Authors:** Luana Moraes Campos, Luciane Donida Bartoli Miot, Mariângela Esther Alencar Marques, Hélio Amante Miot

**Affiliations:** a Department of Dermatology and Radiotherapy, Faculdade de Medicina de Botucatu, Universidade Estadual Paulista, Botucatu, SP, Brazil; b Service of Pathology, Departamento de Patologia, Faculdade de Medicina, Universidade Estadual Paulista, Botucatu, SP, Brazil

**Keywords:** Dermatology, Mesotherapy, Therapeutics

## Abstract

A 28-year-old white female patient presented with multiple erythematous-to-violaceous, painful, suppurative nodules on the buttocks and thighs that appeared after two weeks of mesotherapy with deoxycholate, caffeine, sunflower liposomes, and sinetrol for localized fat. She was treated for atypical mycobacteriosis, but with no satisfactory response after antibiotic therapy. Bacterial, mycobacterial, and fungal culture were all negative. Histopathologic examination of the biopsy showed noninfectious suppurative panniculitis. It resolved after treatment with methotrexate, prednisone, and hydroxychloroquine. This report highlights the rarity of this complication, the importance of its early recognition, and differentiation with atypical fast growing mycobacterioses.

## Case report

**Figure 1:**
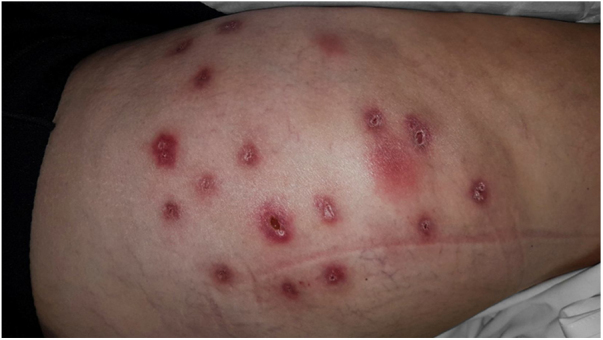
Multiple reddish-violaceous nodules, with suppuration and drainage in areas of mesotherapy application. Thigh and right gluteus.

Female, 28 years of age, white, previously healthy, presenting multiple reddish-violaceous, painful, suppurative nodules on the buttocks and lateral side of the thighs (Fig. 1). She presented after two weeks of mesotherapy with sodium deoxycholate 6%, sunflower liposomes 5%, sinetrol 5%, and 50 mg of caffeine (from a compounding pharmacy); used to treat localized fat deposits, administered by a pharmacist at the patient's office.

**Figure 2:**
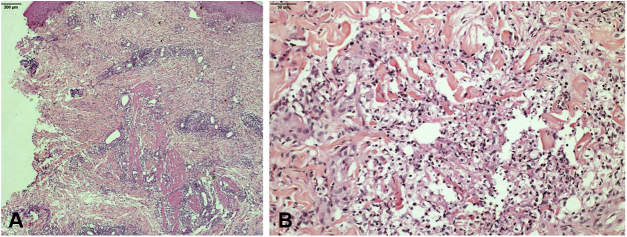
(A) Dermis micrograph showing superficial and deep involvement of the dermis and subcutaneous cellular tissue (Hematoxylin & eosin, x200). (B) Mid-dermis micrograph showing dense lymphohistiocytic infiltrate with neutrophils (Hematoxylin & eosin, x400).

Clarithromycin, ciprofloxacin, doxycycline, and sulfamethoxazole–trimethoprim were used, with no response. Bacterial, mycobacterial, and fungal culture were all negative. Histopathologic examination showed a diffuse lymphohistiocytic inflammatory process with neutrophilic microabscesses affecting the superficial and deep dermis, and subcutaneous cellular tissue (Fig. 2).

**Figure 3:**
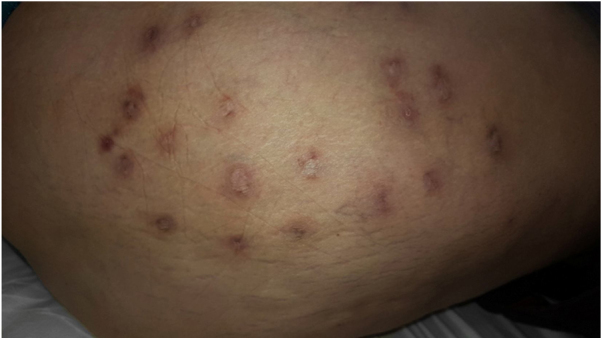
Residual scarring and discreet reddish aspect of nodular lesions after treatment with methotrexate, prednisone, and hydroxychloroquine. Thigh and right gluteus.

In this case, dapsone 100 mg was initiated; however, the patient developed DRESS (drug reaction with eosinophilia and systemic symptoms) after 15 days, being replaced by methotrexate 17.5 mg/week, hydroxychloroquine 400 mg/day, and prednisone 10 mg/day. After two months of treatment, there was substantial improvement of the inflammatory process and evolution to residual cicatricial areas (Fig. 3).

## Discussion

Mesotherapy was described in the 1950s; however, it has been reintroduced as a method of skin rejuvenation and dissolution of adipose tissue, aiming at weight loss, reduced measurements, and reduction of cellulite. In these cases, it induces lipolysis by multiple injections to the subcutaneous adipose tissue of lipolytic substances or detergents such as deoxycholate, phosphatidylcholine, and caffeine.^1^, ^2^, ^3^

Excluding local immediate effects, infection is the most commonly reported complication. There are more than 200 infections reports associated with mesotherapy in the medical literature, all caused by rapidly growing atypical mycobacteria.^4^, ^5^, ^6^

The increased use of this technique, conducted by medical and non-medical professionals, makes it necessary to know possible complications and treatment options. The authors report a case of suppurative noninfectious panniculitis after mesotherapy, a rare event that may result from injection pressure, local trauma, or injected substance types, especially high doses of phosphatidylcholine or deoxycholate.^7^, ^8^

Deoxycholate is the substance most involved, especially in concentrations higher than 5%, as in this case. However, it is not possible to estimate the effect of other substances used or the importance of their association.^7^, ^8^, ^9^, ^10^

The clinical similarity with fast growing mycobacteria infections (*M. fortuitum*, *M. abscessus*, *M. chelonae*, *M. frederiksbergense*, *M. cosmeticum*, *M. peregrinum*, *M. simiae*, *M. immunogenum*, *M. bolleti*, *M. massiliense*) – which can also be inoculated by mesotherapy – requires the search for acid-fast bacilli in the secretion or tissue, which usually shows a large number of bacilli, and whose treatment differs from noninfectious panniculitis.^4^, ^5^, ^6^

There have been only few reports on the treatment of this complication. Until now, no first-choice drug has been established. Some authors have reported the use of corticosteroids and especially dapsone, with good results after two to four months of treatment.^7^, ^8^, ^9^

The present case report aimed to highlight the need to suspect the diagnosis of noninfectious suppurative panniculitis after mesotherapy and the importance of knowing the components and concentrations of injected substances, as well as the value of initiating early appropriate therapy.^10^

## Financial support

None declared.

## Author's contributions

Luana Moraes Campos: Conception and planning of the study; intellectual participation in propaedeutic and/or therapeutic conduct of the cases studied; critical review of the literature; critical review of the manuscript.

Luciane Donida Bartoli Miot: Approval of the final version of the manuscript; elaboration and writing of the manuscript; effective participation in research orientation; intellectual participation in propaedeutic and/or therapeutic conduct of the cases studied; critical review of the literature; critical review of the manuscript.

Mariângela Esther Alencar Marques: Obtaining, analyzing and interpreting the data.

Hélio Amante Miot: Approval of the final version of the manuscript; elaboration and writing of the manuscript; obtaining, analyzing and interpreting the data; intellectual participation in propaedeutic and/or therapeutic conduct of the cases studied; critical review of the literature; critical review of the manuscript.

## Conflicts of interest

None declared.
